# Plant elicitor peptide 1 fortifies root cell walls and triggers a systemic root-to-shoot immune signaling in *Arabidopsis*

**DOI:** 10.1080/15592324.2022.2034270

**Published:** 2022-02-15

**Authors:** Jie Zhang, Yuxi Li, Qixin Bao, Hongbo Wang, Shuguo Hou

**Affiliations:** a School of Municipal & Environmental Engineering, Shandong Jianzhu University, Jinan, China; b College of Biological and Environmental Engineering, Binzhou University, Binzhou, China

**Keywords:** *Arabidopsis*, plant elicitor peptide 1, callose, lignin, jasmonic acid

## Abstract

Plant immunity is initiated by cell surface-localized receptors upon perception of pathogen-derived microbe or pathogen-associated molecular patterns (MAMPs/PAMPs), damage/danger-associated molecular patterns (DAMPs), and phytocytokines. Different patterns activate highly overlapping immune signaling at the early stage but divergent physiological responses at the late stage. Here, we indicate that plant elicitor peptide 1 (Pep1), a well-known DAMP, induces lignin and callose depositions, two types of late immune responses for strengthening the plant cell wall. Pep1-induced lignin and callose depositions in *Arabidopsis* root rely on early signaling components for Pep1 perception and signaling propagation. The phytohormone jasmonic acid and ethylene differently regulate the Pep1-regulated cell wall consolidation. Pep1 application in root also triggers a systemic immune signaling in shoot, and reactive oxygen species (ROS) is essential for the signaling communication between root and shoot. Collectively, the study reveals that Pep1 strengthens cell walls in root and triggers a systemic immune signaling from root to shoot.

## Introduction

Sessile plants are constantly attacked by numerous pathogenic microorganisms in phyllosphere and rhizosphere and have evolved intricate immune mechanisms to defend against these pathogens. One signaling pathway of plant immunity is triggered by a large number of cell surface-localized pattern recognition receptors (PRRs), which perceive microbe-associated molecular patterns (MAMPs), plant-derived damage-associated molecular patterns (DAMPs), and immunological phytocytokines, leading to the activation of pattern-triggered immunity (PTI) and plant broad-spectrum resistance to pathogens.^1^^,^^2^ In aerial plant tissues, local pathogen infections usually trigger resistance to secondary pathogen attacks in distal tissues, a phenomenon called systemic acquired resistance (SAR).^3^ Accumulation of plant hormone salicylic acid (SA) is a hallmark of SAR and is required for the systemic upregulation of defense-related genes.^4^ To activate SAR, a mobile signal(s) is also generated in the locally infected tissues and then transported systemically to the distal tissues. Several signaling entities, including methyl SA, lipid transfer protein DEFECTIVE IN INDUCED RESISTANCE 1 (DIR1), azelaic acid, glycerol-3-phosphate, pipecolic acid, and N-hydroxy-pipecolic acid (NHP), have been identified as candidate mobile signals.^4–6^ Analogous to SAR, plants are also capable to activate another kind of systemic immunity, called induced systemic resistance (ISR), which is primed by plant growth-promoting bacteria and fungi in rhizosphere and enhance defense against a broad range of pathogens and insect herbivores in phyllosphere.^7^ Researches indicated ISR plays similar but different signaling mechanisms with SAR.^7^ For instance, both SAR and ISR require hormonal regulations. However, ISR, unlike SAR, seems to mainly relies on the hormone signaling pathways of jasmonic acid (JA) and ethylene (ET), but not SA.^7^ In addition, it remains greatly enigmatic what long-distance signals are produced by roots and systemically translated to mediate enhanced defense in foliar tissues.^8,9^

Plant elicitor peptide 1 (Pep1), a well-known DAMP, is a 23-amino acid long peptide derived from the carboxyl end of the propeptide, PROPEP1.^10^ Upon MAMP perception or cell damage, vacuolar membrane-resident PROPEP1 is processed by type II metacaspases to release Pep1 into the apoplasts, where it is perceived by extracellular LRR domain of two homologous LRR-RK family receptors, PEP RECEPTOR 1 (PEPR1) and PEPR2, resulting in the association of PEPRs with BRASSINOSTEROID INSENSITIVE 1 (BRI1)-associated receptor kinase 1 (BAK1)/SERK3 and SERK4 and consequent activation of the receptor complex.^11–18^ The activated receptor complex in turn activates conserved cytoplasmic signaling events, including phosphorylation of the receptor-like cytoplasmic kinases (RLCKs) *BOTRYTIS*-INDUCED KINASE 1 (BIK1), BIK1 close homolog AVRPPHB SUSCEPTIBLE1 (PBS1)-LIKE 1 (PBL1), and mitogen-activated protein kinases (MAPKs), the rise of apoplastic reactive oxygen species (ROS) and cytosolic calcium, and extensive transcriptional reprogramming.^2,19–22^ The induction of the early immune signaling and transcriptional reprogramming leads to late physiological responses and final plant resistance to pathogens. In addition, local application of Pep in leaves also activates a phytohormone-mediated immune signaling in systemic parts of plants.^23^

Phytohormones, including SA, JA, and ET, play crucial roles in the regulation of plant immune signaling. ISOCHORISMATE SYNTHASE 1/SALICYLIC ACID-INDUCTION DEFICIENT 2 (ICS1/SID2) is a key enzyme responsible for SA biosynthesis during pathogen infections in *Arabidopsis*. SA regulates gene transcriptional reprogramming mainly through NPR1 (NON-EXPRESSOR of PATHOGENESIS-RELATED GENES 1) and contributes to local resistance and SAR against biotrophic pathogens.^3^ JA is synthesized and conjugated with isoleucine to form the active hormone JA-Ile, which is perceived by CORONATINE INSENSITIVE 1 (COI1) and induces COI1 association with JASMONATE ZIM-DOMAIN (JAZ) proteins. The interaction between COI1 and JAZs causes the degradation of JAZs and relieves their repression on bHLH transcription factors, MYC2, MYC3 and MYC4, resulting in the activation of a subset of downstream wound response and resistance to herbivorous insects.^24–26^ ET signaling is mediated by the central regulator ETHYLENE INSENSITIVE 2 (EIN2) and two primary transcription factors downstream of EIN2, EIN3 and its closest homolog EIN3-LIKE 1 (EIL1).^27,28^ JAZs also act as transcriptional repressors of JA-responsive genes by binding to EIN3/EIL1.^29^ JA and ET thus act synergistically in regulating plant resistance to necrotrophic pathogens through EIN3/EIL1. However, ET suppresses JA-MYC-mediated expression of wounding-responsive and herbivory-inducible genes and attenuates JA-regulated plant defense against herbivores.^24,30–32^

Plant cell walls are protective barriers against pathogens. The deposition of callose and lignin during plant–pathogen interactions is thought as a strengthening of the plant cell wall and contributes to plant resistance to pathogens.^33^ Callose is a β(1,3)-glucan polymer. Callose deposition represents as a typical marker response of PTI as well as a part of penetration resistance.^34^ Callose deposition in *Arabidopsis* leaves upon MAMP treatments or fungal penetration relies on the callose synthase POWDERYMILDEW RESISTANT 4 (PMR4).^35^ ROS, ET signaling, and glucosinolate metabolites are required for MAMP-induced callose deposition in leaves.^35^ Lignin is an aromatic polymer, one of the most important secondary metabolites. Lignin metabolism has been widely reported to be relevant to plant resistance to pathogens and pests, as well as the tolerance to some abiotic stresses.^36^ However, with molecular mechanisms revealing cell wall lignification in plants in response to pathogen infections, little has been known.

Compared to the phyllosphere, the rhizosphere is a microbe-rich environment. This makes plant roots more vulnerable to pathogen attacks. However, the mechanism of root immunity, in contrast to that of well-studied leaf immunity, is poorly understood. Previous studies indicated that Pep1 significantly suppresses root growth, activates early immune responses in roots, and contributes to plant resistance to the root pathogen *Pythium irregulare*.^10,37–39^ Recently, it was revealed that Pep1 activates cell type-specific immunity networks in root, which is distinct from flg22 does.^40^ In this study, we unveiled that Pep1 treatments lead to the deposition of callose and lignin in root. The Pep1-induced callose and lignin depositions in root rely on conserved PTI signaling components and is oppositely regulated by JA and ET. MAMP flg22 pretreatments increase the Pep1-induced callose and lignin deposition. In addition, we also indicate that Pep1 treatments in root trigger SA and ROS-dependent systemic immune responses in shoot.

## Results and discussion

### 1. Pep1 induces callose and lignin in shoots and roots

**Figure 1.**
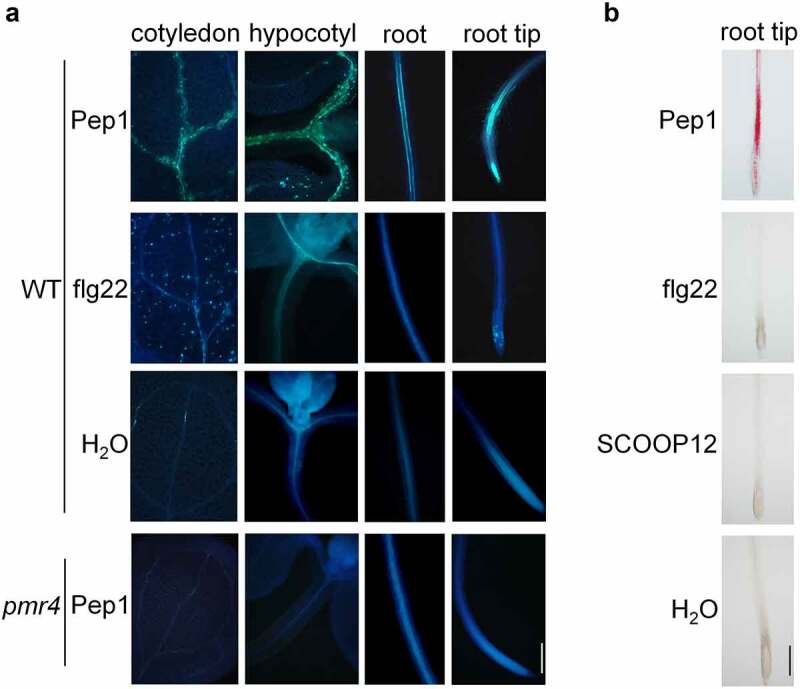
**Pep1 induces callose and lignin depositions in roots and shoots**. (a) Pep1 induces callose deposition in the vasculature of shoot and root. (b) Pep1 induces lignin deposition in the vasculature of shoot and root. One-week-old plate-grown seedlings of WT or *pmr4* mutants were treated with H_2_O, 1 μM Pep1, 1 μM SCOOP12, or 1 μM flg22 for 24 hours, followed by callose (A) or lignin staining (B). At least ten seedlings for each treatment were detected with similar results. Bar = 200 μm. All experiments were repeated three times with similar results.

As representatives of MAMP and DAMP peptides, flg22 and Pep1 employ overlapping early immune signaling components but trigger different late immune responses and plant physiological changes. To further determine the difference of immune activation at a late stage between flg22 and Pep1, we examined callose deposition in *Arabidopsis* seedlings upon treatments with Pep1 and flg22. We found that flg22 is capable to strongly induce callose deposition in mesophyll cells of cotyledons^35^ but only marginally induces the callose deposition in the elongation zone (EZ) of roots as reported previously (Figure 1a).^35,41^ In contrast, Pep1 substantially induces callose deposition in vascular system of cotyledons, hypocotyls, and roots. The EZ of root tips shows the strongest induction of callose deposition (Figure 1a, Supplementary figure 1). No callose deposition was detected in the *pmr4* mutant that lacks a functional callose synthase required for various stimuli-triggered callose disposition in leaves (Figure 1a). Moreover, a phytocytokine SCOOP12 recently reported to strongly activate root immune responses,^42^ also cannot induce root callose deposition (Supplementary figure 2).

To explore furtherly the late response of plant roots to Pep1, we examined lignin production in roots upon 24-hour treatment with the both immune peptide elicitors. We found that Pep1, but not flg22 and SCOOP12, clearly induced lignin deposition in vasculature of roots, especially in EZ and the junction of primary and lateral roots, as indicated by pink color after phloroglucinol staining (Figure 1b). The induction of lignin deposition is in a dose-dependent manner (Supplementary figure 3). Collectively, our result demonstrated that Pep1 specifically triggers callose and lignin depositions in vasculature of roots, especially in EZ of root tips. Soil-borne phytopathogens, such as *Fusarium* and *Verticillium* wilt pathogens, preferentially infect EZ of roots and colonize the plant vascular system.^43^ *PROPEP1* and paralogous *PROPEP2* and *PROPEP3* are highly induced by MAMPs, pathogens, and insect herbivores.^10,23,44–46^ Pep peptides also play a conserved function in pathogen/herbivore resistance across different plant species.^22,44,46–48^ Transgenic *Arabidopsis* plants overexpressing *PROPEP1* and *PROPEP2* exhibit an enhanced resistance to the root pathogen *Pythium irregulare*.^10^ Therefore, Pep1 signaling may be employed by plants to defend against diverse pathogens and herbivores by strengthening cell walls in roots.

### 2. Pep1-induced callose and lignin deposition relies on in PEPR1/2, BAK1/SERK4, BIK1/PBL1, and AGB1

**Figure 2.**
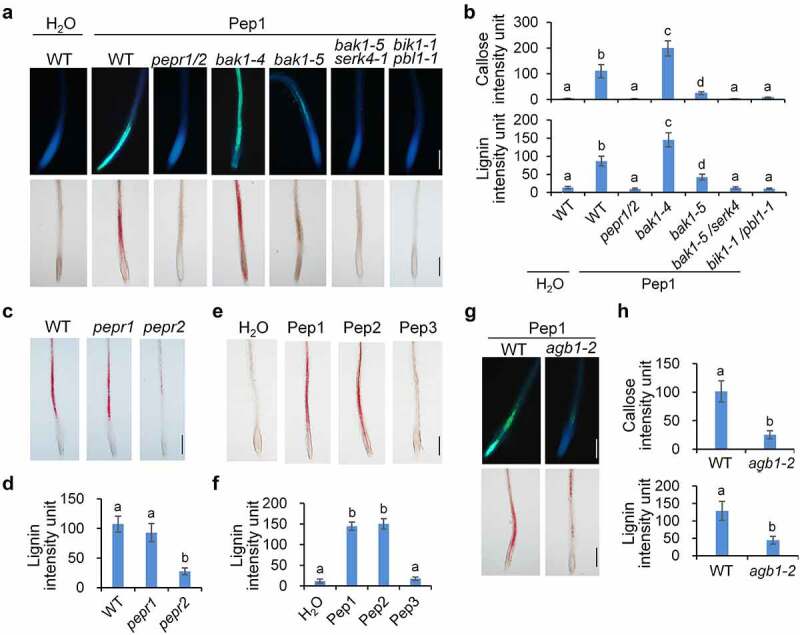
**Pep1-induced callose and lignin deposition relies on PEPR1/2, BAK1/SERK4, BIK1/PBL1 and AGB1**. (a and b) Pep1 induces callose and lignin depositions through PEPR1/2, BAK1/SERK4, and BIK1/PBL1. (c and d) PEPR2 plays a major role in the Pep1-induced lignin disposition. (e and f) Pep1, Pep2, but not Pep3 induce lignin disposition in root tips.(g-h) Pep1 induces callose and lignin deposition through AGB1. One-week-old plate-grown seedlings of indicated genotypes were treated with H_2_O or 1 μM Pep1 for 24 hours, followed by callose or lignin staining. Quantification data of callose and lignin in b, d, f, and h were indicated as means of intensity unit from each repeat. Significant differences were shown by different letters (Student’s *t*-test, *n* ≥ 8). The experiment was repeated three times with similar results. Bar = 200 μm.

To investigate the signaling mechanism used by Pep1 for the induction of callose and lignin depositions, we examined the cell wall modifications in roots of a series of mutants of genes required for the Pep1-triggered signaling pathway. It was indicated that *pepr1*/*pepr2*, the mutant of Pep1 receptor genes, is completely insensitive to Pep1 for the induction of callose and lignin (Figure 2a, B). Like *pepr1/pepr2, pepr2* seedlings are also unable to deposit lignin in roots upon Pep1 treatments, but *pepr1* seedlings have no discernible difference from WT seedlings for the induction of callose and lignin (Figure 2c, D), suggesting that PEPR2 is responsible for the Pep1-induced lignin disposition in roots. Consistent with this, Pep2, which is perceived by both PEPR1 and PEPR2, also strongly activates lignin deposition, whereas Pep3, which is only perceived by PEPR1,^13^ cannot induce lignin formation (Figure 2e, f). The Pep1-induced callose and lignin depositions are greatly impaired in *bak1-5*, the *bak1* allele with a point substitution in the kinase domain, and completely abolished in *bak1-5*/*serk4* (Figure 2a). These results are consistent with a previous report that BAK1 positively regulates Pep signaling.^49^ In contrast, the *bak1-4* knockout mutant does not reduce but significantly enhances the Pep1-induced callose and lignin deposition. Callose and lignin in *bak1-4* upon Pep1 treatment are not enclosed in root tips but in vasculature of the whole root (Figure 2a, B). It was reported that loss of BAK1 but not catalytic inactivation reinforces Pep activation of immune responses and cell death,^18^ suggesting that the Pep1-induced callose and lignin depositions and cell death response may share a similar mechanism. Upon Pep1 perception, PEPR1/PEPR2 specifically interacts with BIK1 and PBL1 to mediate Pep1-induced defenses.^22,44^ Pep1-induced callose and lignin deposition is completely abolished in *bik1*/*pbl1* plants (Figure 2a, b). The heterotrimeric G-protein β subunit, AGB1, has been shown to modulate PTI signaling and plant cell wall integrity responses.^50–52^ We found that Pep1-induced callose and lignin deposition in roots are also substantially attenuated in *agb1-2* mutants (Figure 2g, h), suggesting that heterotrimeric G proteins may mediate Pep1-induced response in roots.

### 3. ROS and calcium signaling regulate Pep1-induced callose and lignin deposition

**Figure 3.**
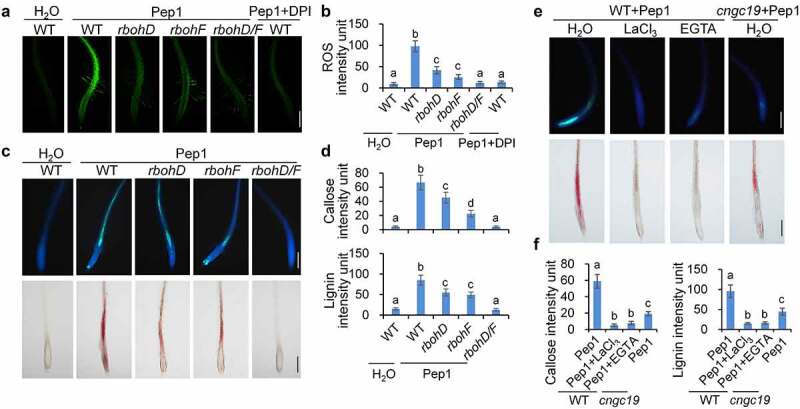
**ROS and calcium are required for Pep1-induced callose and lignin depositions**. (a and b) Pep1 induces H_2_O_2_ production through RBOHD and RBOHF in roots. One-week-old plate-grown seedlings of indicated genotypes were treated with H_2_O, 1 μM Pep1, or a combination of 1 μM Pep1 and 10 μM DPI, H_2_O_2_ in root tips were detected through H_2_DCF-DA staining. Bars = 200 μm. (c and d) Pep1-induced callose and lignin depositions through RBOHD/F. One-week-old plate-grown seedlings of indicated genotypes were treated with 1 μM Pep1 or a combination of 1 μM Pep1 and 10 μM DPI, callose and lignin were stained post 24 hours induction. Bars = 200 μm. (e and f) Pep1-induced lignin deposition is regulated by calcium channels. One-week-old plate-grown seedlings of indicated genotypes were treated with 1 μM Pep1 or a combination of 1 μM Pep1 and 1 mM LaCl_3_ or 10 mM EGTA, callose or lignin were stained post 24-hour treatment. Quantification data of ROS, callose, and lignin in b, d, and f, were indicated as means of intensity unit from each repeat. Significant differences were shown by different letters (Student’s *t*-test, *n* ≥ 8). The experiment was repeated three times with similar results.

ROS and calcium are two crucial early signaling components for Pep1 signaling pathway. Pep1-induced ROS production and cytosolic Ca^2+^ increase downstream of BIK1/PBL1.^22,53,54^ Moreover, Pep1-triggered ROS production is more pronounced than that of flg22 in roots.^39^ The NADPH oxidase RbohD and RbohF have been indicated to mediate the Pep1-induced ROS in roots,^54^ which is suppressed by diphenylene iodonium (DPI), a chemical inhibitor of NADPH oxidases (Figure 3a, B). We found that the Pep1-induced callose and lignin depositions remain almost intact in *rbohD* but greatly reduced in *rbohF* and completely lost in the *rbohD/F* double mutants (Figure 3c, D), suggesting that RbohD/RbohF-mediated ROS production is required for Pep1-induced callose and lignin depositions in roots. Consistent with this, DPI suppresses the Pep1-induced callose and lignin depositions in roots (Figure 3c, D). The phytocytokine SCOOP12 is able to induce stronger ROS production in roots than Pep1 and flg22 (Supplementary figure 4A),^42,55,56^ however, it does not induce lignin and callose depositions in roots (Figure 1b, Supplementary figure 2). We found that the Pep1- and SCOOP12-induced ROS production exhibits different tissue specificity. The Pep1-induced ROS distributes across different cell types of roots. In contrast, SCOOP12-indcued ROS seems to be exclusively produced in the epidermis of roots (Supplementary figure 4B). The tissue-specific induction of ROS by Pep1 and SCOOP12 agrees with the localization of Pep1 receptors PEPRs in diverse type of root cells and SCOOP12 receptor MIK2 in root epidermis.^42,57–59^ It also suggests that the vasculature-localized factor besides ROS may play a critical role in the induction of callose and lignin. Moreover, we indicated that flg22 pretreatment enhances Pep1-induced callose and lignin depositions (Supplementary figure 4C). It was reported that flg22 pretreatment enhances the Pep1-triggered ROS production,^60^ further supporting that ROS is essential for the Pep1 induction of callose and lignin deposition.

Pep1 and SCOOP12 also induce stronger Ca^2+^ influx in roots than flg22 does (Supplementary figure 5A),^61^ and the Pep1 induction of cytosolic calcium increase is blocked by LaCl_3_, an inhibitor Ca^2+^ channel, and EGTA, a Ca^2+^ chelator (Supplementary figure 5B). LaCl_3_ and EGTA significantly block Pep1-induced callose and lignin deposition (Figure 3e, f), suggesting that the cytosolic Ca^2+^ increase is essential for the Pep1-induced root responses. It has recently been reported that the calcium channel CYCLIC NUCLEOTIDE GATED CHANNEL 19 (CNGC19) is required for the Pep1-induced cytosolic Ca^2+^ increase in *Arabidopsis* seedlings.^62^ In line with this, Pep1-induced callose and lignin depositions are substantially reduced in the roots of *cngc19* null mutants compared to WT seedlings (Figure 3e, f). Together, ROS production and cytosolic calcium increase are essential for Pep1-induced callose and lignin depositions.

### 4. JA and ET oppositely regulate Pep1-induced callose and lignin deposition

**Figure 4.**
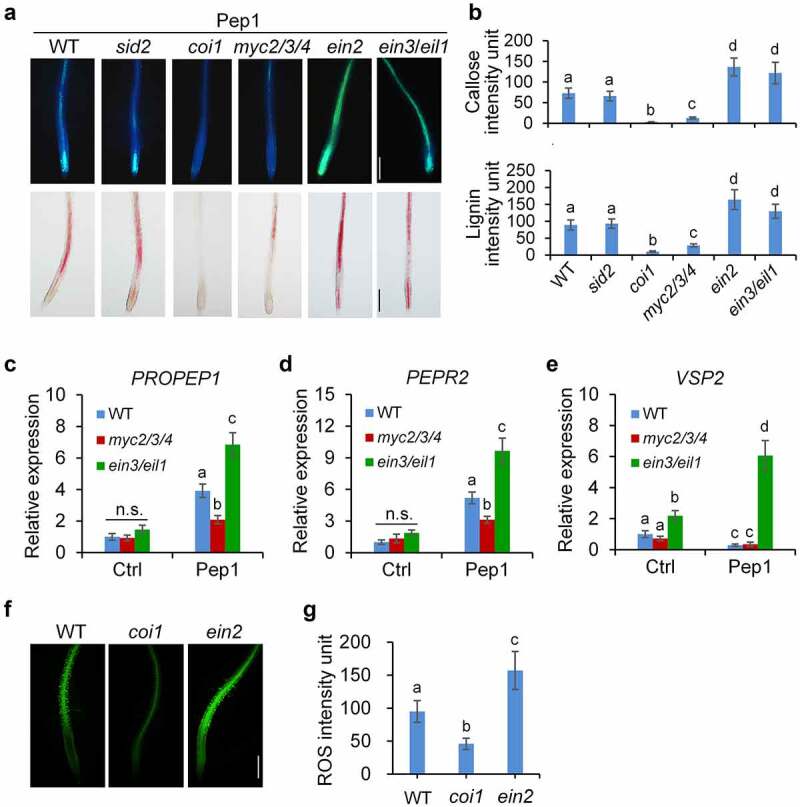
**JA and ET oppositely regulate Pep1-induced callose and lignin depositions**. (a and b) Pep1-induced callose and lignin depositions are abolished in *coi1* but enhanced in *ein2*. One-week-old plate-grown seedlings of indicated genotypes were treated with H_2_O or 1 μM Pep1 for 24 hours, followed by callose or lignin staining. Bar = 200 μm. (c–e) Pep1-regulated expression of *PROPEP1* (d), *PEPR2* (e) and *VSP2* (f) is attenuated in *myc2/3/4* but enhanced in *ein3/eil1*. One-week-old seedlings of indicated genotypes were treated with 1 μM Pep1, mRNA was isolated for RT-qPCR analysis of gene transcription levels 3 (d and e) or 24 (f) hours post treatment. Different letters indicate a significant difference with others (*P* < .01, n.s., no significant differences, Student’s *t*-test, *n* = 3). (f and g) Pep1-induced H_2_O_2_ production is attenuated in *coi1* but enhanced in *ein2*. One-week-old plate-grown seedlings were treated with 1 μM Pep1, H_2_O_2_ in root tips were detected through H_2_DCF-DA staining. Bar = 200 μm. Quantification data of ROS, callose, and lignin in b, d, and f were indicated as means of intensity unit from each repeat. Significant differences were shown by different letters (Student’s *t*-test, *n* ≥ 8). The experiments were repeated three times with similar results.

To test whether phytohormone signaling pathways are also involved in the Pep1-induced callose and lignin depositions in roots, we examined the Pep1-induced responses in *coi1, ein2*, and *sid2* mutants, which disrupts JA, ET, and SA signaling, respectively. We found that the Pep1-induced callose and lignin depositions are not changed in *sid2* mutants but completely abolished in *coi1* mutants and greatly enhanced in *ein2* mutants in comparison with WT seedlings (Figure 4a, b). These results suggest that JA signaling promotes but ET signaling suppresses the Pep1-induced callose and lignin depositions in roots. We also detected other Pep1-induced root phenotypes. We found that Pep1-induced root growth inhibition is attenuated in *coi1* but not in *ein2* (Supplementary figure 6A). It also agrees with previous findings that Pep1 regulate seedling growth independent of EIN2^22^. Pep1 induces root hair formation in root tips through auxin and ET signaling.^59^ In line with this, Pep1 induction of root hair formation is abolished in *ein2* but not in *coi1* (Supplementary figure 6B). These results suggest that Pep1-induced callose and lignin depositions may be relevant to root growth inhibition but irrelevant to root hair formation.

JA perception by COI1 leads to the activation of MYC2/3/4-mediated signaling pathway responsible for wound response and defense against insect herbivores and EIN3/EIL1-mediated signaling pathway required for root development and resistance to necrotrophic fungi.^31,63^ Pep1-induced callose and lignin deposition is significantly impaired in *myc2*/*3*/*4* mutants but robustly induced in *ein3*/*eil1* double mutants (Figure 4a, b), suggesting that the COI1-MYC2/3/4 branch of JA signaling pathway is required for the Pep1-induced callose and lignin depositions, which is antagonized by the EIN3/EIL1 pathway. It was reported that ET but not JA signaling is required for flg22-induced callose deposition in leaves and roots,^35,41^ suggesting that the signaling mechanism employed by Pep1 and flg22 for the induction of callose deposition is different.

**Figure 5.**
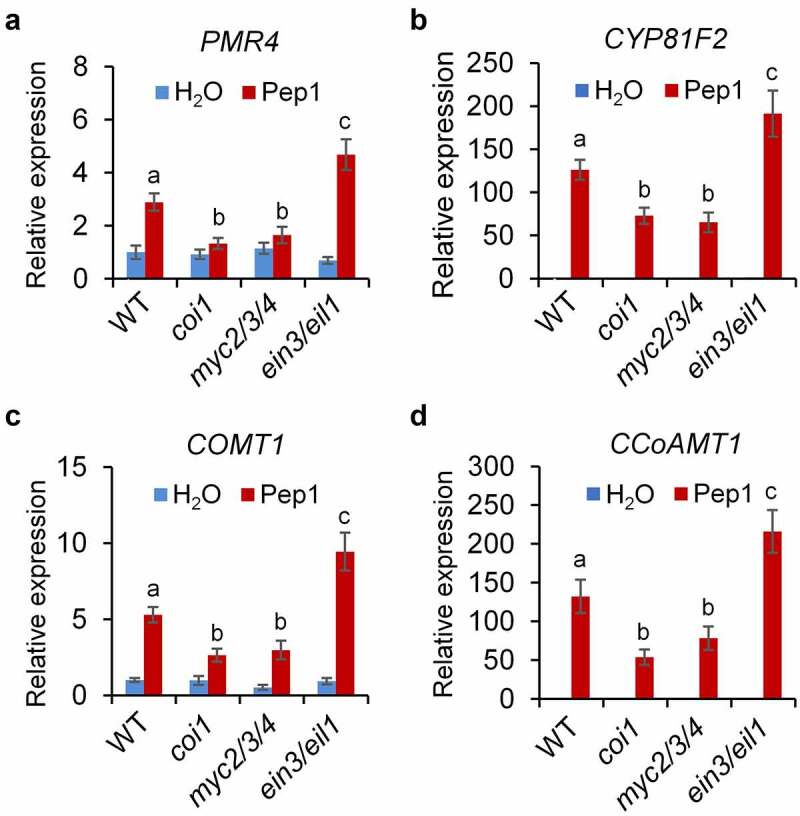
**JA and ET signaling oppositely regulate Pep1-induced expression of genes involved in callose and lignin biosynthesis**. One-week-old seedlings of indicated genotypes were treated with 1 μM Pep1, mRNA was isolated for RT-qPCR analysis of gene transcription levels 6 hours post treatment. Different letters indicate a significant difference with others (*P* < .01, Student’s *t*-test, *n* = 3). The experiments were repeated three times with similar results.

To decipher how JA and ET signaling regulate the Pep1 responses in roots, we analyzed the expression of *PROPEP1* and *PEPR2* which are upregulated by Pep1.^57^ We found that the Pep1-induced *PROPEP1* and *PEPR2* expression levels are lower in *myc2/3/4* but higher in *ein3/eil1* than that of WT plants (Figure 4c, d). Pep1 suppresses the expression of *VEGETATIVE STORAGE PROTEIN* 2 (*VSP2)*, a marker gene of JA-MYC2/3/4 signaling, in WT and *myc2/3/4* mutants. However, Pep1 upregulates *VSP2* expression in *ein3/eil1* (Figure 4e). These results suggest that EIN3/EIL1 antagonizes MYC2/3/4-mediated Pep1 signaling. Moreover, we found that Pep1-induced ROS production in root is weaker in *coi1* but stronger in *ein2* than that of WT seedlings (figure 4f, g). This data is consistent with a previous report that JA signaling pathway is required for the Pep1-induced ROS production.^60^ CYTOCHROME P450, FAMILY 81 (CYP81F2), a cytochrome P450 monooxygenase responsible for indole glucosinolate O-methyltransferases, and the callose synthase PMR4 are required for MAMP-induced callose deposition.^35^ Caffeoyl CoA O-methyltransferase 1 (CCoAOMT1) and caffeate 3-O-methyltransferase 1 (COMT1) are involved in the synthesis of lignin.^64,65^ We indicated that Pep1 is able to upregulate the expression of *PMR4, CYP81F2, CCoAOMT1*, and *COMT1* in roots (Figure 5a-d). The upregulation of these genes by Pep1 is also attenuated in *myc2/3/4* but enhanced in *ein3*/*eil1* mutants (Figure 5a-d), suggesting that JA and ET oppositely regulate the expression of genes required for Pep1-induced callose and lignin biosynthesis. Pep1 was reported to induce the ethylene-responsive gene *PDF1.2* and repress the MYC2-dependent branch of JA-responsive gene *VSP2*.^10,22,44^ It was recently reported that Pep1 was shown to regulate distinct transcriptional factor-mediated gene networks in different root cell types.^40^ Therefore, Pep1 might differently activate MYC2/3/4 and EIN2/EIL1 pathways in a cell/tissue-specific manner. The activation of MYC2/3/4 pathway responsible for callose and lignin depositions may play a dominant role in the vasculature of roots. However, the activation of EIN3/EIL1 and consequent suppression of MYC2/3/4 pathway may dominantly work in other tissues.

### 5. Pep1 generates systemic immune signaling from roots to shoots

**Figure 6.**
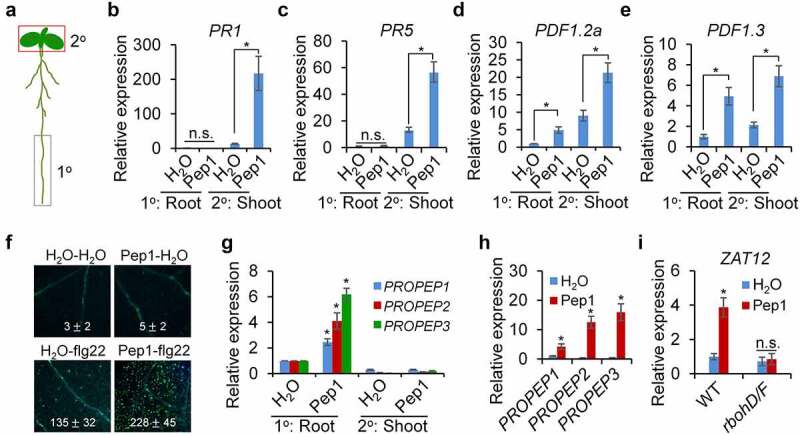
**Pep1 triggers a systemic immune signaling from the root to the shoot**. (a) A schematic diagram for the definition of local (1°) and systemic (2°) tissues. Root in the black box was treated with peptide elicitor and shoot in red box was used for gene expression analysis. (b–e) Pep1 application in roots induces systemic *PR1* (b), *PR5* (c), *PDF1.2* (d), and *PDF1.3* (e)expression in shoot. (f) Pretreatment of Pep1 in roots enhances flg22-induced callose deposition in shoots. Roots of 10-d-old plate-grown seedlings were pretreated with 1 μM Pep1 or H_2_O for 24 hours, then the shoot of seedlings was treated with 1 μM flg22 or H_2_O for another 24 hours before callose deposition staining. Callose deposits in cotyledons were measured using ImageJ. Data are shown as mean ± s.e.m (*n* = 8). (g) Pep1 application in roots induces *PROPEP* expression in roots but not in shoots. (h) Pep1 application in shoots induces *PROPEP* expression in shoots. (i) Pep1 application in roots induces *ZAT12* expression in shoot RBOHD/F-dependent manner. (b–e, g–i) The lower half roots of 1-week-old seedlings grown on 1/2MS plate were treated with 1 μM Pep1 or H_2_O for 24 hours, roots were then cut off for seedlings for mRNA isolation and RT-qPCR analysis of gene transcription levels. Different letters indicate a significant difference with others (*P* < .01, n.s., no significant differences, Student’s *t*-test, *n* = 3). The experiments were repeated three times with similar results.

Pep1 strongly triggers callose deposition in the vasculature of root and shoot (Figure 1a, Supplementary figure 1), and it promotes us to determine whether Pep1 mediates a signaling communication between the root and the shoot. We first treated Pep1 in the lower half of the root and explored callose deposition in the root and the shoot (Figure 6a). We found that callose deposition was only induced in the root but not in the hypocotyl and the cotyledon by Pep1 application in the root (Supplementary figure 7). We further analyzed the expression of some Pep1 responsive immune-related genes. We found that local application of Pep1 in the root upregulates shoot expression of *PATHOGENESIS-RELATED PROTEIN* 1 (*PR1*) and *PR5*, the marker genes of SA signaling pathway (Figure 6b, c). Likewise, Pep1 application in the root also induces shot expression of *PDF1.2a* and *PDF1.3*, the marker genes of JA signaling pathway. However, the expression of *PDF1.2a* and *PDF1.3*, but not *PR1*, is locally upregulated by Pep1 in the root (Figure 6d, e). Therefore, Pep1 locally activates JA signaling in roots and systemically induces SA and JA signaling in shoots. It has been reported that local Pep1 application in leaves triggers *PR1* and *PDF1.2* upregulation in systemic leaves,^23^ suggesting a similarity for the signaling communications between leaf-to-leaf and root-to-shoot. In addition, pretreatment of Pep1 in roots significantly enhanced flg22-induced callose deposition in shoots (figure 6f), implying a Pep1 function in plant immune priming. Serval peptide signals, such as CEP1 and CLE25, have been reported to be as mobile signals in mediating signaling communications between the root and the shoot.^66,67^ To investigate if Pep1 is a mobile signal, we analyzed the *PROPEP* expression in roots and shoots when both parts are respectively applied with Pep1 peptides. We found that Pep1 can locally upregulate the expression of *PROPEP1, PROPEP2*, and *PROPEP3* in both shoots and roots (Figure 6g, h). However, root application of Pep1 is unable to upregulate the systemic expression of these genes in shoots (Figure 6g). These results suggest that Pep1 may not play as a mobile signal traveling from the root to the shoot. This is consistent with a previous report that Pep1 does not travel from leaf to leaf.^23^ It has been reported that ROS generated in roots upon multiple stimuli can be systemically transported to shoots.^68,69^ We found that Pep1 application in roots also induced the expression of *ZAT12*, a ROS-responsive gene, in shoots, and the Pep1-induced *ZAT12* expression in abolished in *rbohD/F* (Figure 6i). Therefore, ROS may also mediate the Pep1-triggered long-distance signaling from the root to the shoot.

## Conclusion

**Figure 7.**
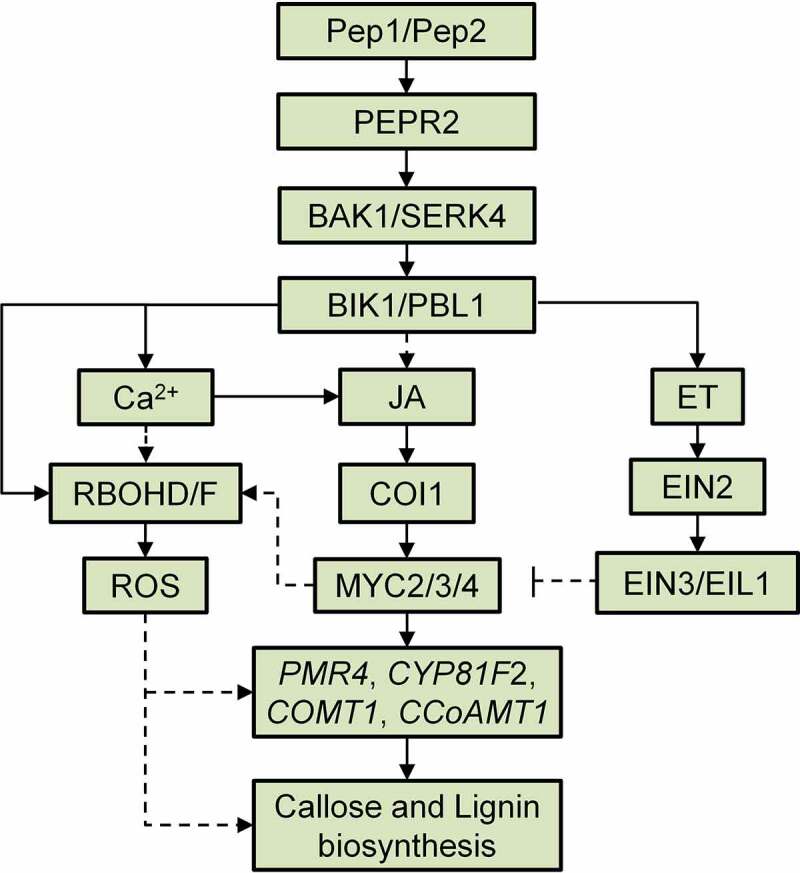
**A proposed model of the Pep1-induced callose and lignin deposition in root**. Pep1 and Pep2 are perceived by PEPR2 and transduce a signaling through BAK1/SERK4 and BIK1/PBL1, which leads to cytosolic Ca^2+^ increase and RBOHD/F-mediated ROS production. Ca^2+^ and ROS signals mediate the deposition of callose and lignin in roots. JA-COI1-MYC2/3/4 and ET-EIN2-EIN3/EIL1 signaling pathways oppositely regulate Pep1-induced ROS production and ROS-mediated callose lignin deposition. JA-COI1-MYC2/3/4-ROS may contribute to the expression of Pep and PEPR2 through a positive feedback regulation.

The plant cell wall is a natural physical barrier to pathogens. Plants are able to strengthen cell walls in tissues where they are attacked by pathogens. Perception of various PAMPs and DAMPs triggers cell wall modifications, such as callose and lignin deposition. In this study, we indicated that Pep1, different from the PAMP flg22 and the phytocytokine SCOOP12, specifically induces callose and lignin depositions in *Arabidopsis* roots. It was found that Pep1-regulated callose and lignin depositions share a greatly overlapped signaling pathway downstream of Pep1 perception by its receptor PEPR2. Some early immune signaling components in PTI signaling pathway, including BAK1/SERK4, BIK1/PBL1, ROS, cytosolic Ca^2+^, and G protein are required for the Pep1-induced cell wall responses in roots (Figure 7). Phytohormones, JA and ET play opposite roles in the regulation of Pep1-induced callose and lignin deposition. The opposite roles of JA and ET is correlated with their regulation of Pep1-induced ROS production and the expression of genes involved callose and lignin biosynthesis in root (Figure 7). In addition, we also indicated that Pep1 application in root triggers a ROS-mediated systemic signaling in shoots, implying that Pep1 may regulate a root-to-shoot signaling communication. Overall, this study unveiled a signaling mechanism for Pep1 regulation of plant root callose and lignin depositions and discovered a Pep1-mediated signaling communication between root and shoot, which may advance our understanding of the plant immune regulation.

## Material and Methods

### Plant material and growth conditions

The *Arabidopsis thaliana* accession Columbia-0 (Col-0) was used as wild-type (WT). The *bak1-4, bak1-5, bik1/pbl1, rbohD, rbohF, rbohD/F, pmr4, coi1-2, sid2-2, ein2-1, ein3-1/eil1-1, agb1-2*, and *cngc19* mutants were described previously.^70–73^ The *pepr1, pepr2*, and *pepr1-2*/*pepr2-2* mutants were shared by Dr Zhi Qi (Inner Mongolia University, China), *myc2/3/4* mutant seeds were shared by Dr Haitao Cui. Seedlings used for histochemical assays and gene expression analysis, were grown on half-strength Murashige and Skoog^1/2MS^ plates containing 0.5% (w/v) sucrose, 0.75% (w/v) agar, and 2.5 mM MES, pH 5.8, in a growth chamber at 20–23°C, 50% humidity, and 75–100 μE m^−2^ s^−1^ light with a 12-hour light/12-hour dark photoperiod.

### Total RNA isolation, cDNA synthesis, and RT-qPCR

Total RNA was extracted from 10-d-old seedlings grown on ½MS plates using TRIzol reagent (Invitrogen). One microgram of total RNA was reverse-transcribed to synthesize the first-strand cDNA with M-MuLV Reverse Transcriptases (Thermo Fisher Scientific) and oligo(dT) primers following by RNase-free DNase I (Thermo Fisher Scientific) treatment. RT-qPCR analyses were performed on a QuantStudio™ 3 Real-Time PCR Detection System (Thermo Fisher Scientific) using Faster Universal SYBR® Green Master (Roche) and gene-specific primers following the standard protocol. The expression of each gene was normalized to the expression of *UBQ10*. The primers used for RT-qPCR are listed in Supplementary Table 1.

### Callose staining

Callose deposits were stained as described previously.^41^ Roots or shoots of 10-d-old seedlings grown on ½MS plate were dip-incubated with or without 1 µM Pep1 for 24 hours, followed by fixation in a 3:1 ethanol:acetic acid solution for 6 hours. The fixative was changed three times to ensure both thorough fixing and clearing of the tissues, which is essential for good callose detection in the roots. Seedlings were rehydrated in 70% ethanol for 2 hours, 50% ethanol for an additional 2 hours, and water overnight. After two washes with water, seedlings were treated with 10% NaOH for 10 minutes to make the tissues transparent. After three washes with water and one wash with 150 mM K_2_HPO_4_ (pH 9.5), seedlings were incubated in aniline blue staining solution (150 mM K_2_HPO_4_, pH 9.5, 0.01% aniline blue (Sigma-Aldrich)) for 1 hour and then washed with 150 mM K_2_HPO_4_ (pH 9.5). The seedlings were mounted on slides, and callose deposits were observed immediately using the Olympu BX53 microscope equipped with DP74 CCD camera under UV (excitation, 390 nm; emission, 460 nm).

### Lignin staining

Root tips of 10-d-old seedlings grown on ½MS plate were treated with 1 μM peptide on plates. Lignin in root tips was stained with phloroglucinol-HCl 24 hours after treatments as described previously with some modifications.^74^ Seedlings on plates was first incubated with 10 µM HCl for 5 minutes followed by treated with equal volume of 5% (w/v) phloroglucinol for another 10 minutes. The seedlings were then moved onto a glass slide and kept for 2 minutes for oxidation before adding phloroglucinol solution and covering with a cover slip. The seedlings were photographed using the Olympu BX53 microscope equipped with DP74 CCD camera under white light.

### H_2_O_2_ staining

Root tips of 10-d-old seedlings grown on ½MS plate were treated with H_2_O or 1 μM Pep1 on plates for 24 hours. H_2_O_2_ in root tips was detected with 2′,7′-dichlorofluorescein diacetate (H_2_DCF-DA) (Sigma-Aldrich, St. Louis, MO, USA) staining. In brief, the seedlings were incubated in 25 μM H_2_DCF-DA solution for 10 minutes in darkness. After three washes with water, seedlings were photographed under fluorescence microscopy (BX53, Olympus, Tokyo, Japan) equipped with DP74 CCD camera (excitation, 460 nm; emission, 520 nm).

### Measurement of ROS production

ROS burst was determined by a luminol-based assay. Twenty roots of 1-week-old seedlings grown on ½MS plates were incubated in 200 μL ddH_2_O overnight in a 96-well plate. Then, ddH_2_O was replaced by 200 µL of reaction solution containing 50 µM of luminol, and 10 µg/mL of horseradish peroxidase (Sigma-Aldrich) supplemented with or without 100 nM or 1 μM peptide. Luminescence was measured immediately after adding the solution with a luminometer (Glomax 20/20 n, Promega) with a 30-second interval for 15 minutes. The total values of ROS production were indicated as means of the relative light units (RLU).

### Measurement of cytosolic Ca^2+^ concentration

Cytosolic Ca^2+^ concentration was measured as described previously.^42^ Twenty roots of 1-week-old seedlings expressing *p35S::Aequorin* grown vertically on ½MS plates were put into a 96-well plate containing 200 μL solution with 1 mM KCl and 1 mM CaCl_2_. Aequorin was reconstituted by treating the seedlings with coelenterazine-h (Promega, Beijing, China) in the dark overnight at a final concentration of 10 µM. Luminescence was measured with a luminometer (Glomax 20/20 n, Promega) with a ten-second interval for 6 minutes. The values for cytosolic Ca^2+^ concentrations were indicated as means of RLU.

## Supplementary Material

Supplemental Material
