# Supplementary material for: Plant elicitor peptide 1 fortifies root cell walls and triggers a systemic root-to-shoot immune signaling in Arabidopsis
Source: Plant Signal Behav. 2022 Feb 15;17(1):2034270. doi: 10.1080/15592324.2022.2034270 (PMC9176251; doi:10.1080/15592324.2022.2034270)
Supplement: Supplemental Material [file KPSB_A_2034270_SM0290.zip › psb Supplementary_marterials-1.docx]

**Supplementary materials**


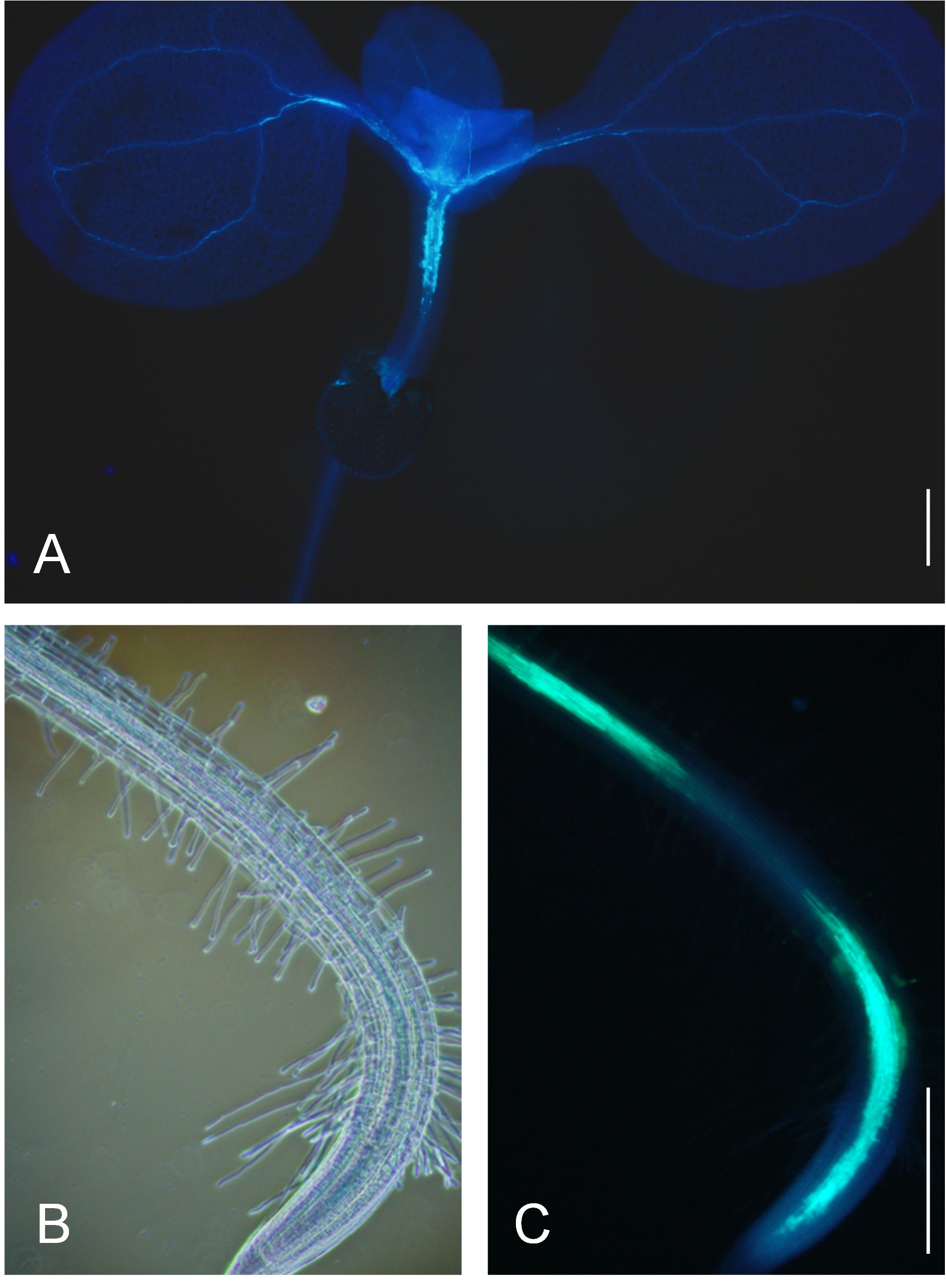


**Supplementary figure 1.** Pep1 induces callose deposition in the vasculature of roots and shoots. One-week old plate-grown seedlings were treated with 1 μM Pep1for 24 hour followed by callose staining. (A) Pep1-induced callose deposition in hypocotyls and main veins of cotyledons. (B, C) Pep1-induced callose deposition in vascular of roots. (B) image under the white light; (C) image under the fluorescent light. The experiments were repeated three times with similar results. Bar = 200 μm.


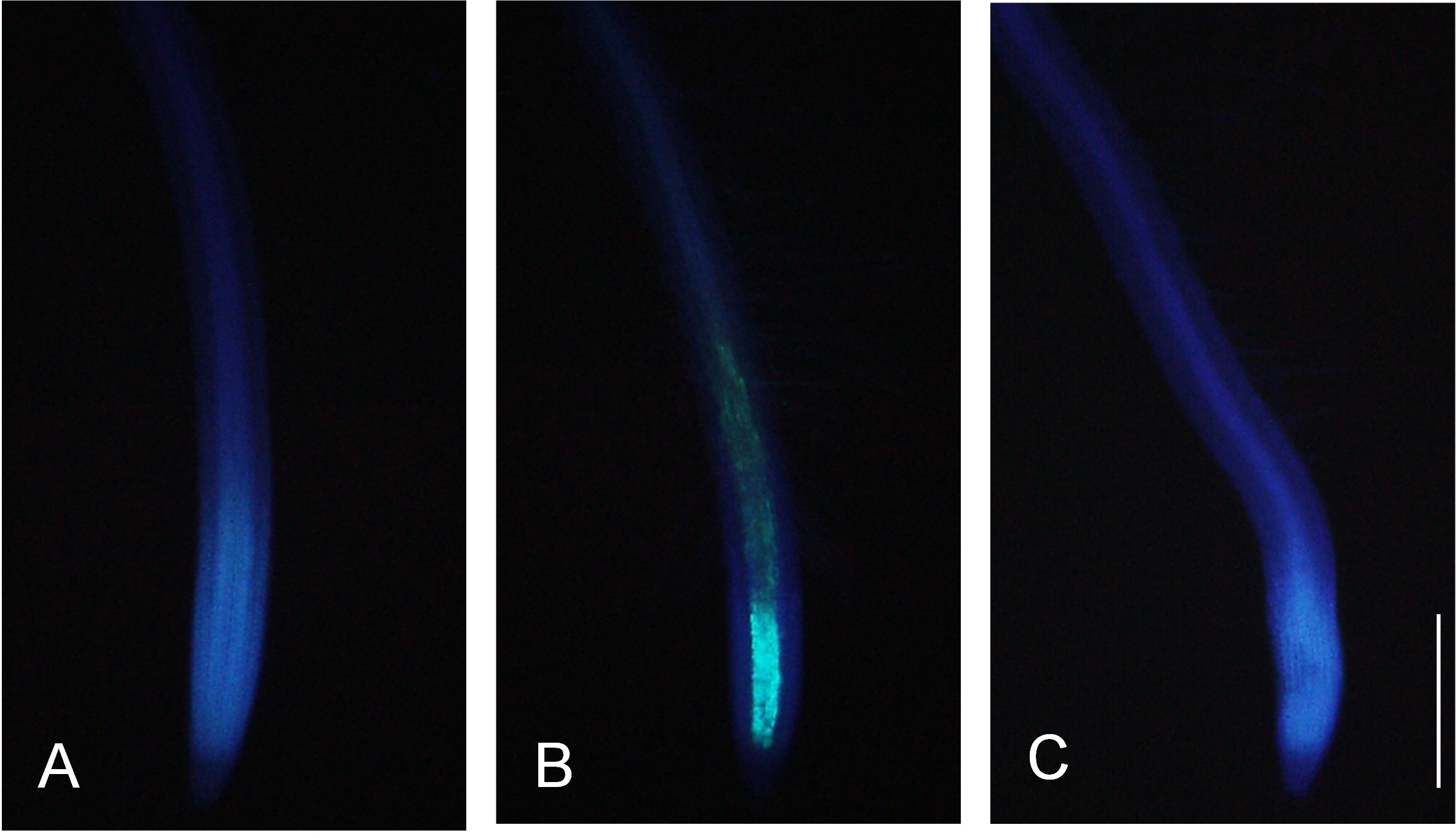


**Supplementary figure 2.** SCOOP12 does not induce callose deposition in roots. One-week old plate-grown seedlings were treated with H_2_O (A), 1 μM Pep1 (B), or SCOOP12 (C) for 24 hours followed by callose staining. Bar = 200 μm.


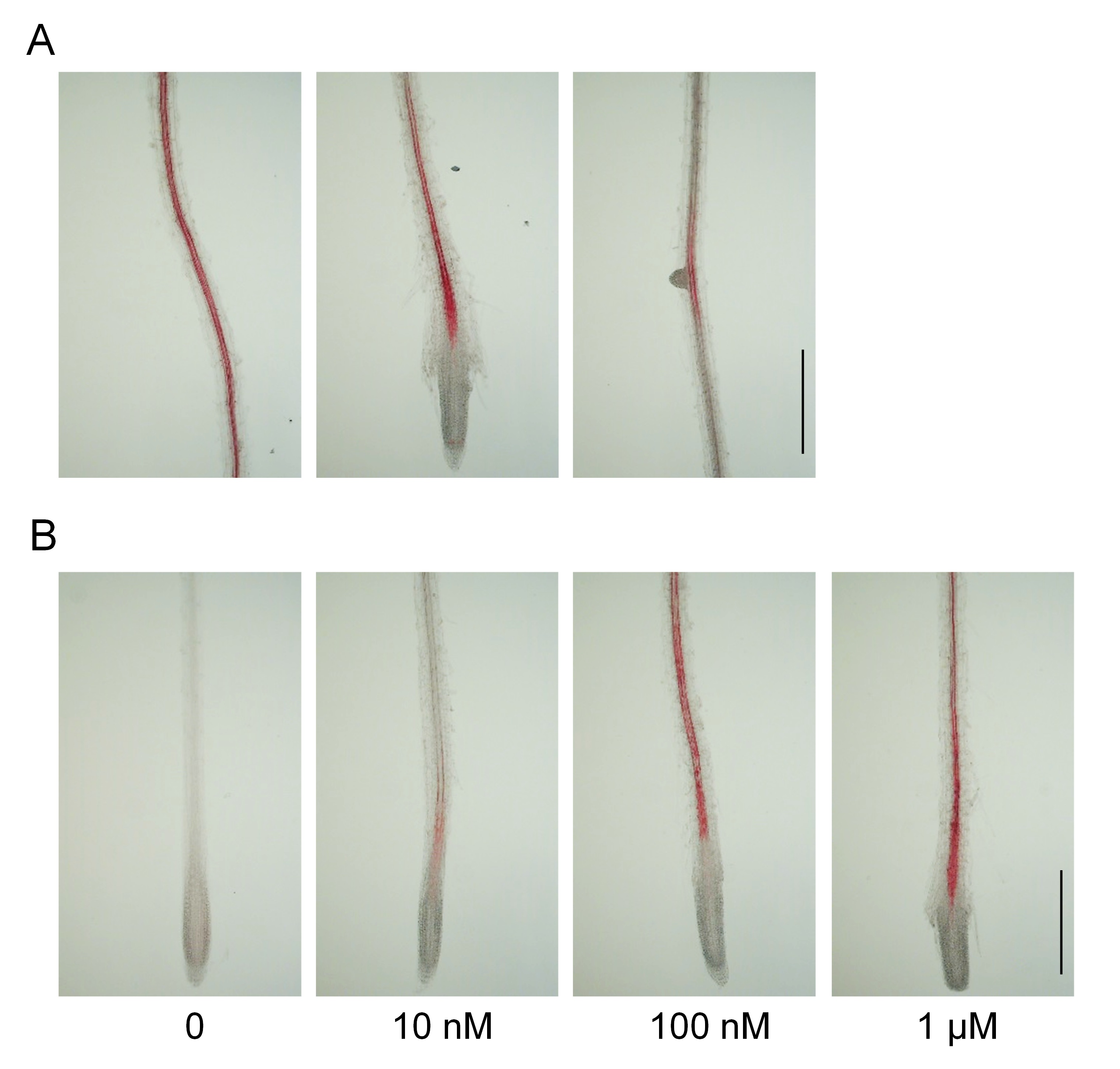


**Supplementary figure 3.** Pep1 induces lignin deposition in roots in a dose-dependent manner. One-week old plate-grown seedlings were treated with 1 μM Pep1 (A) or Pep1 with indicated concentration (B) for 24 hours followed by callose staining. Bar = 200 μm.


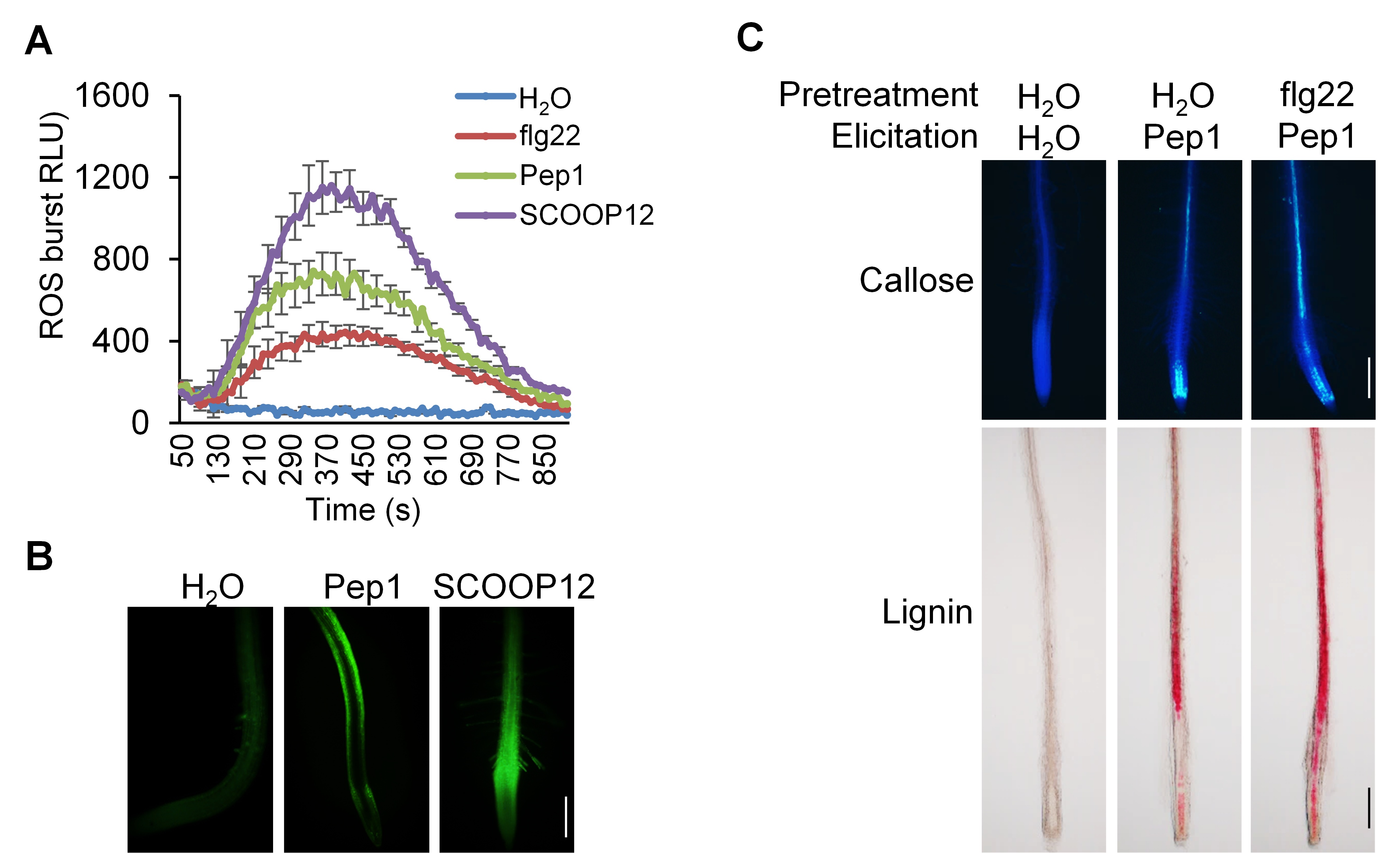


**Supplementary figure 4.** Pep1, SCOOP12, and flg22 differentially induce ROS production in roots, and flg22 pretreatment enhances Pep1-induced lignin and callose deposition.

(A) Pep1 and SCOOP12 induced stronger ROS production in root than flg22 does. The values for ROS production were indicated as means of RLU from five repeats.

(B) Pep1 and SCOOP12 induce tissue-specific H_2_O_2_ production in roots. One-week old plate-grown WT seedlings were treated with H_2_O, 1 μM Pep1, or 1 μM SCOOP12, H_2_O_2_ in root tips were detected through H_2_DCF-DA staining. Bars = 200 μm.

(C) Flg22 pretreatment enhances Pep1-induced lignin and callose depositions. Root of one-week old plate-grown seedlings were pretreated with H_2_O or 1 μM flg22 for 3 hours on plates, followed by treatments with H_2_O or 1 μM Pep1 for 24 hours before callose staining. Bar = 200 μm.

All experiments were repeated twice with similar results.


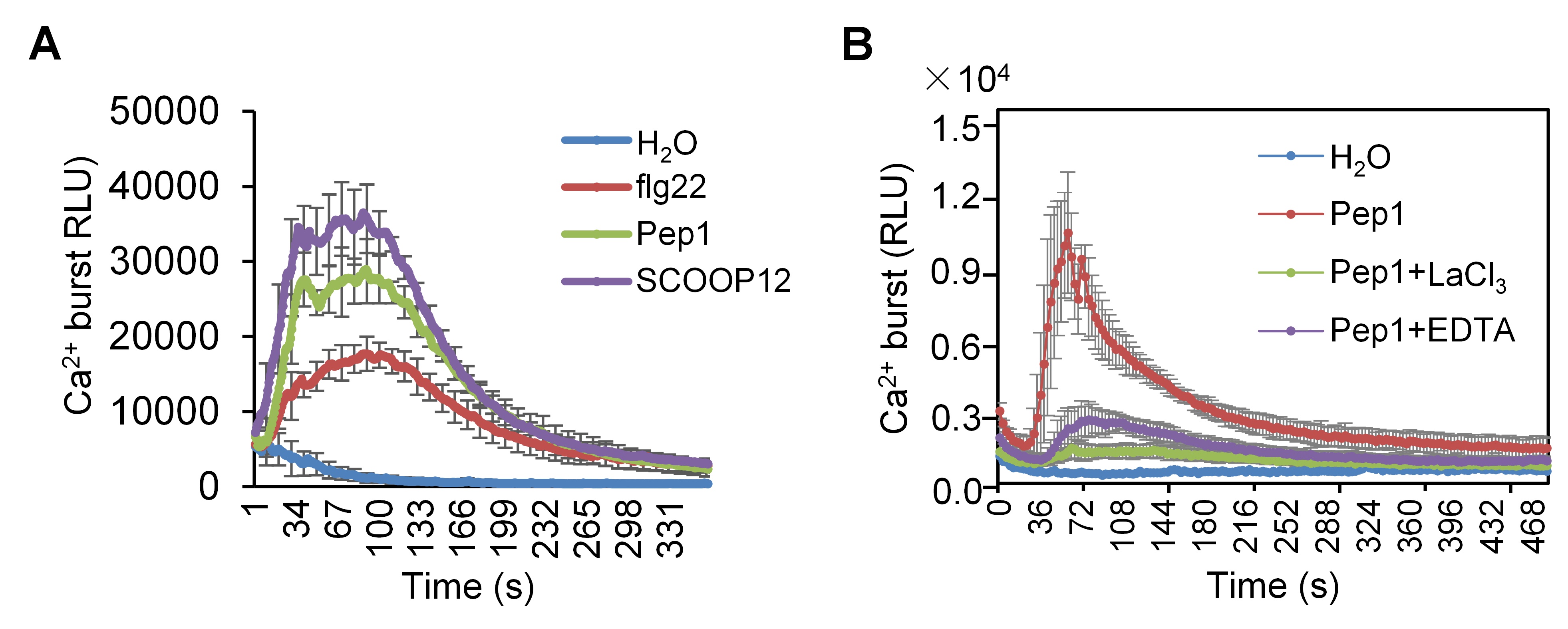


**Supplementary figure 5.** Pep1 induces the increase of cytosolic calcium in roots.

(A) Pep1 and SCOOP12 induce stronger cytosolic calcium increase in roots than flg22 does.

(B) Pep1 induction of cytosolic calcium increase is blocked by the calcium channel blocker LaCl_3_ and calcium-chelating agent ETDA.

One-week old aequorin expressed transgenic seedlings were induced with H_2_O, 1 μM Pep1, 1 μM SCOOP12, 1 μM flg22, or a combination of 1 μM Pep1 and 1 mM LaCl_3_ or 10 mM EDTA. Cytosolic calcium concentration was detected as relative luminescence unit (RLU).


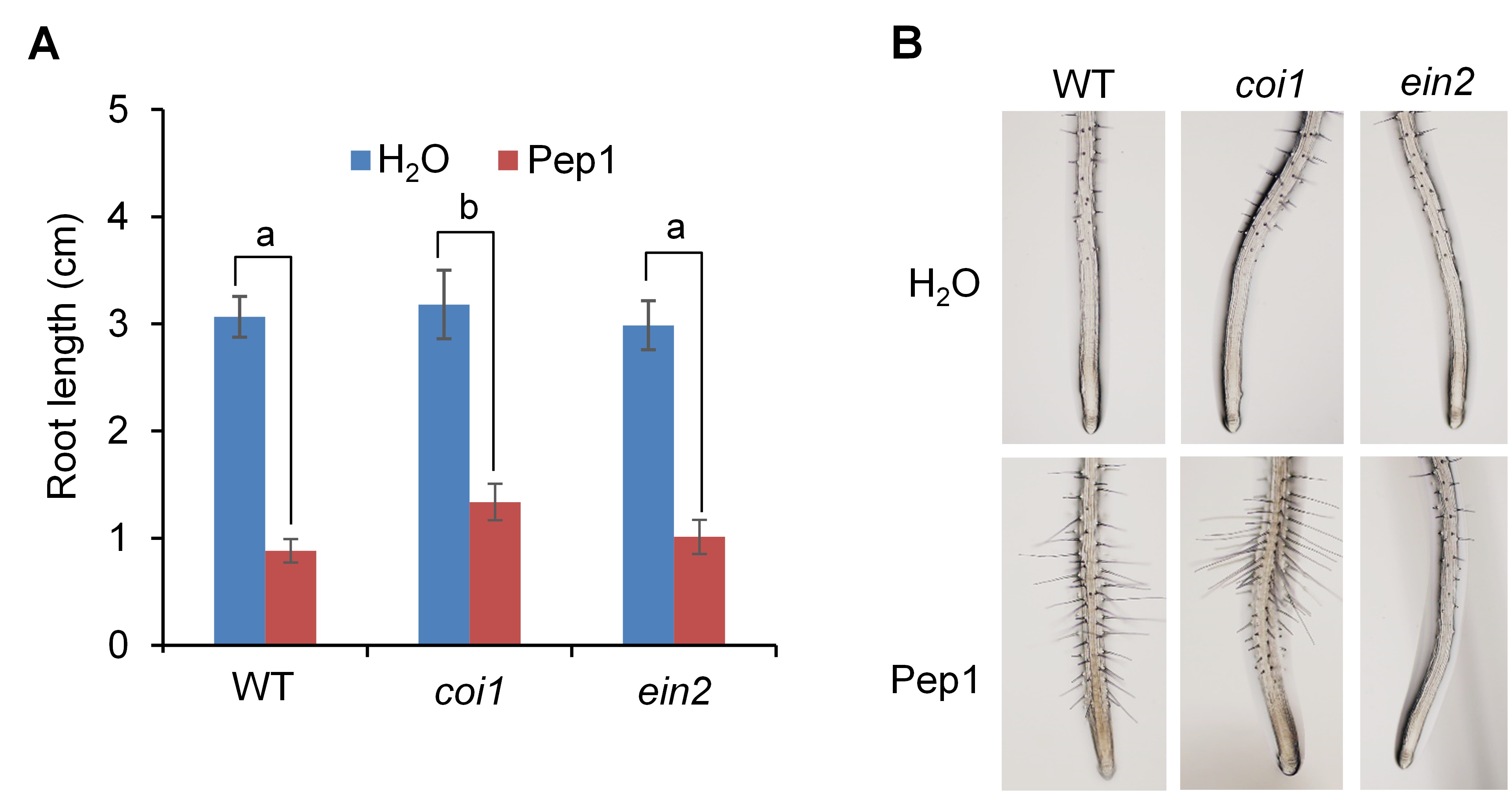


Supplementary figure 6. Pep1 suppresses root growth and induces root hair formation by acting on hormone signaling.

(A) JA but not ET signaling is required for Pep1 suppression on root growth. Three-day old plate-grown seedlings were transferred onto 1/2 MS plate containing 1 μM Pep1 for another five days before measurement of root length. Significant differences were shown by different letters (Student’s *t*-test, *n* ≥ 8).

(B) ET but not JA signaling is required for Pep1-induced root hair formation. Five-day old plate-grown seedlings were treated with 1 μM Pep1 on root tips, followed by imaging 24 hours later.

All experiments were repeated twice with similar results.


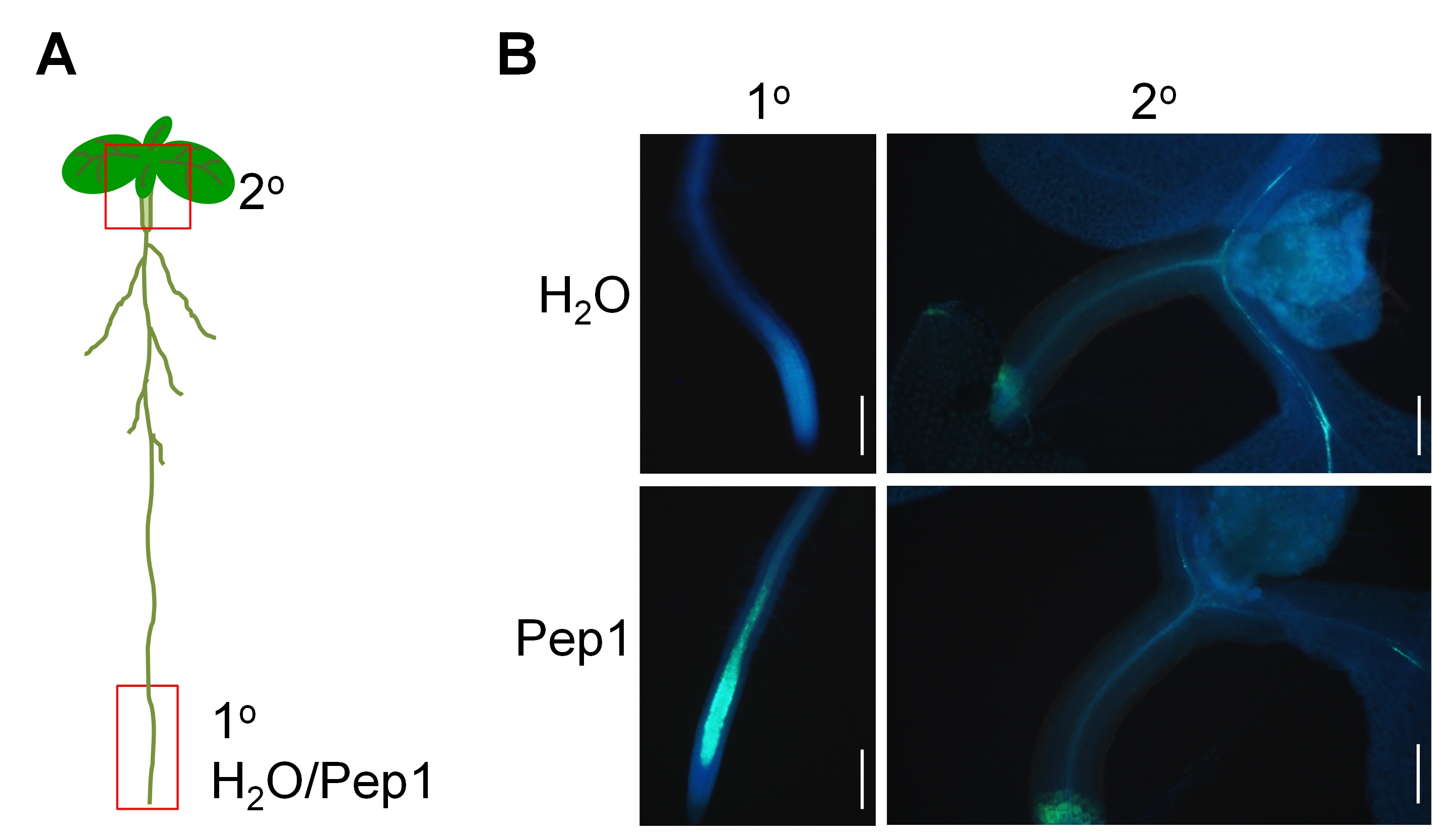


**Supplementary figure 7.** Pep1 application in root does not induce callose deposition in shoot. (A) A schematic diagram for the definition of local (1^o^) and systemic (2^o^) tissues (B) Pep1 induces local root callose deposition but not systemic shoot callose deposition. Root of one-week old plate-grown seedlings were treated with H_2_O, or 1 μM Pep1 for 24 hours, followed by callose staining. The experiments were repeated three times with similar results. Bar = 200 μm.

**Supplementary Table 1. Primers used in this study.**

| **Gene name** | **Gene ID** | **Forward primer (5'→3')** | **Reverse primer (5'→3')** |
| --- | --- | --- | --- |
| *UBQ10* | AT4G05320 | AGATCCAGGACAAGGAAGGTATTC | CGCAGGACCAAGTGAAGAGTAG |
| *PROPEP1* | AT5G64900 | GAGCTATCTTGAAATGCCTTGG | CTAACTTTCTCCTTCCCCCTTT |
| *PROPEP2* | AT5G64890 | AAGACTATGGTGTTGCTCTGAA | ATCCCTTTTCTTTGATTTGGCC |
| *PROPEP3* | AT5G64905 | ACGATTCCTCTCTTGAAGTGTT | GCTTAGTCTTGTTCTTCCCTCT |
| *PEPR2* | AT1G17750 | CAATGAGTTTAGGCCTGAATCG | TATCTTCAAGTCTGACTCGCTC |
| *VSP2* | AT5G24770 | TCAGTGACCGTTGGAAGTTGTG | GTTCGAACCATTAGGCTTCAATATG |
| *PMR4* | AT4G03550 | CAAGCAATGAAGACTTCTACCG | ATTACTGAAGAAAGCAATCCGC |
| *CYP81F2* | AT5G57220 | CTTCTTGCAGATTTGACGTTCA | TCCTTGTTATGAACCTGGTCTC |
| *COMT1* | AT5G54160 | GGCTTTAAAATCCGCCTTAGAG | CAGGATTTTTGGTCGGAAGTTT |
| *CCoAMT* | AT1G67980 | ATTGGTGAACGACAAATGTGAG | CCCCAACCTTCACCAATTTTAG |
| *PR1* | AT2G14610 | CTAAGAGGCAACTGCAGACT | GTATGGCTTCTCGTTCACAT |
| *PR5* | AT1G75040 | AGGATTTGAATTGACTCCAGGT | CCATCGCCTACTAGAGTGAATT |
| *PDF1.2a* | AT5G44420 | CTTATCTTCGCTGCTCTTGTTC | TGGGAAGACATAGTTGCATGAT |
| *PDF1.3* | AT2G26020 | CTCTTGTTCTCTTTGCAGCTTT | TGGGAAGACATAGTTGCAAGAT |
| *ZAT12* | AT5G59820 | TGTCGTCTGGATTGATGAAGAA | GATTTCTTCAACGTAGTCACCG |
