# Supplementary material for: Plant elicitor peptide 1 fortifies root cell walls and triggers a systemic root-to-shoot immune signaling in Arabidopsis
Source: Plant Signal Behav. 2022 Feb 15;17(1):2034270. doi: 10.1080/15592324.2022.2034270 (PMC9176251; doi:10.1080/15592324.2022.2034270)
Supplement: Supplemental Material [file KPSB_A_2034270_SM0290.zip › Supplementary marterials.pdf]

Supplementary materials

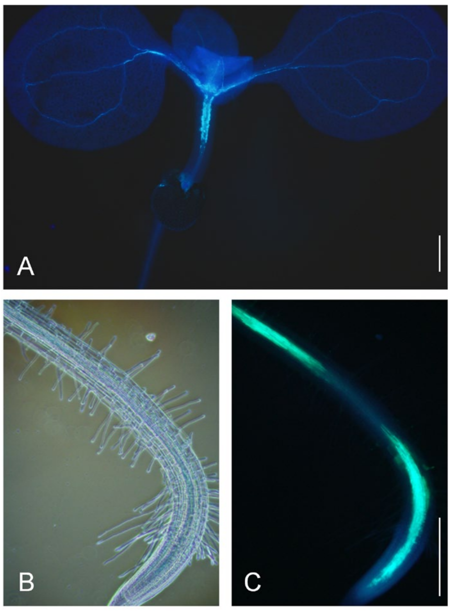

Supplementary figure 1. Pep1 induces callose deposition in the vasculature of roots and shoots. One-week old plate-grown seedlings were treated with 1 μM Pep1for 24 hour followed by callose staining. (A) Pep1-induced callose deposition in hypocotyls and main veins of cotyledons. (B, C) Pep1-induced callose deposition in vascular of roots. (B) image under the white light; (C) image under the fluorescent light. The experiments were repeated three times with similar results. Bar = 200 μm.

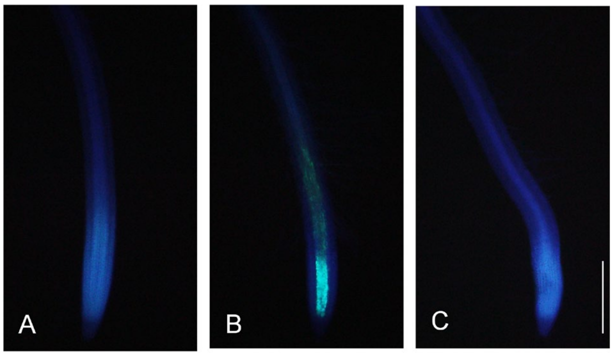

Supplementary figure 2. SCOOP12 does not induce callose deposition in roots. One-week old plate-grown seedlings were treated with H_2O (A), 1 µM Pep1 (B), or SCOOP12 (C) for 24 hours followed by callose staining. Bar = 200 µm.

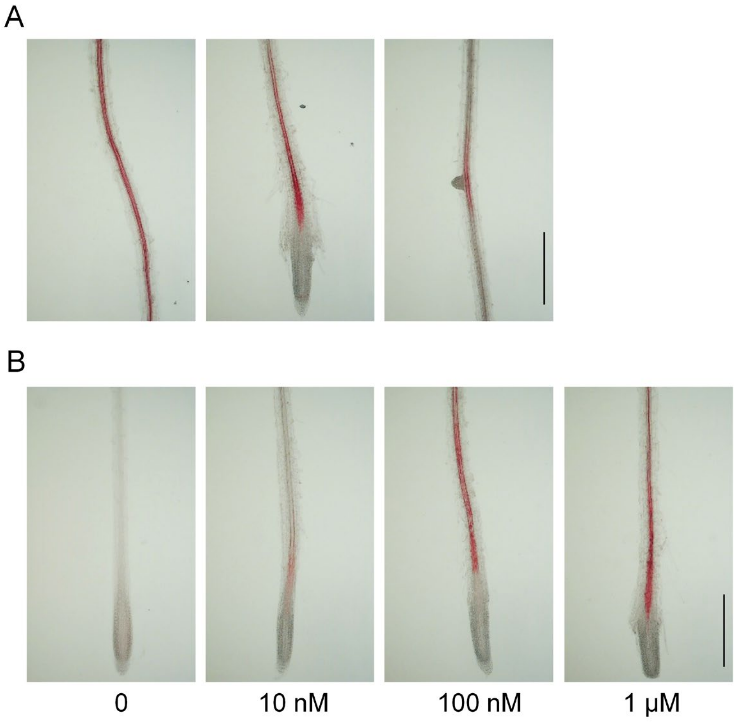

Supplementary figure 3. Pep1 induces lignin deposition in roots in a dose-dependent manner. One-week old plate-grown seedlings were treated with 1 μM Pep1 (A) or Pep1 with indicated concentration (B) for 24 hours followed by callose staining. Bar = 200 μm.

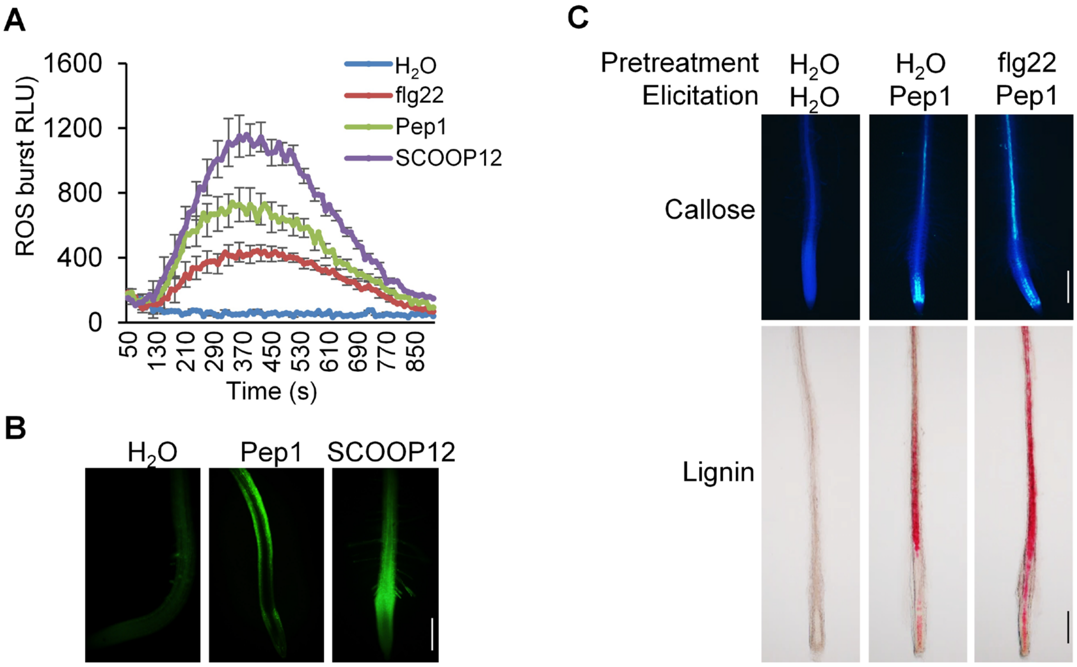

Supplementary figure 4. Pep1, SCOOP12, and flg22 differentially induce ROS production in roots, and flg22 pretreatment enhances Pep1-induced lignin and callose deposition.

(A) Pep1 and SCOOP12 induced stronger ROS production in root than flg22 does. The values for ROS production were indicated as means of RLU from five repeats.

(B) Pep1 and SCOOP12 induce tissue-specific H_2O_2 production in roots. One-week old plate-grown WT seedlings were treated with H_2O, 1 μM Pep1, or 1 μM SCOOP12, H_2O_2 in root tips were detected through H_2DCF-DA staining. Bars = 200 μm.

(C) Flg22 pretreatment enhances Pep1-induced lignin and callose depositions. Root of one-week old plate-grown seedlings were pretreated with H_2O or 1 μM flg22 for 3 hours on plates, followed by treatments with H_2O or 1 μM Pep1 for 24 hours before callose staining. Bar = 200 μm.

All experiments were repeated twice with similar results.

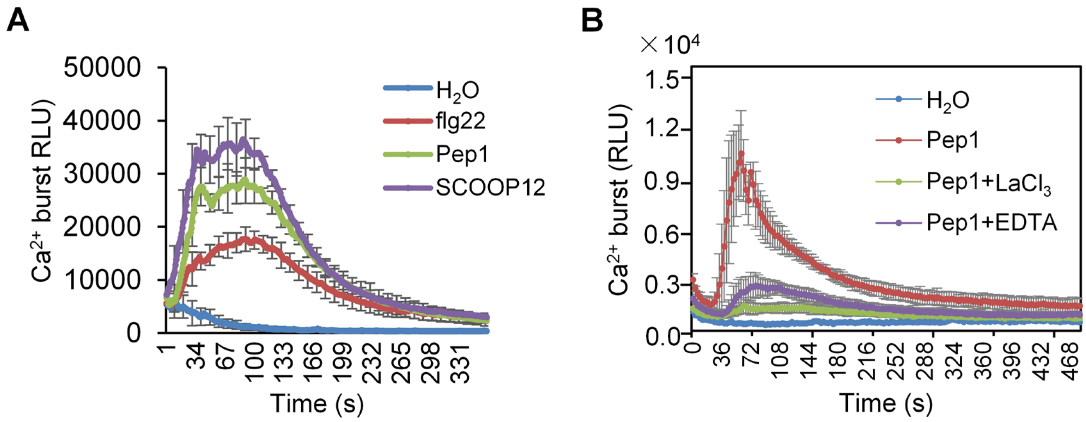

Supplementary figure 5. Pep1 induces the increase of cytosolic calcium in roots.

(A) Pep1 and SCOOP12 induce stronger cytosolic calcium increase in roots than flg22 does.

(B) Pep1 induction of cytosolic calcium increase is blocked by the calcium channel blocker $LaCl_3$ and calcium-chelating agent ETDA.

One-week old aequorin expressed transgenic seedlings were induced with H_2O, 1 μM Pep1, 1 μM SCOOP12, 1 μM flg22, or a combination of 1 μM Pep1 and 1 mM $LaCl_3$ or 10 mM EDTA. Cytosolic calcium concentration was detected as relative luminescence unit (RLU).

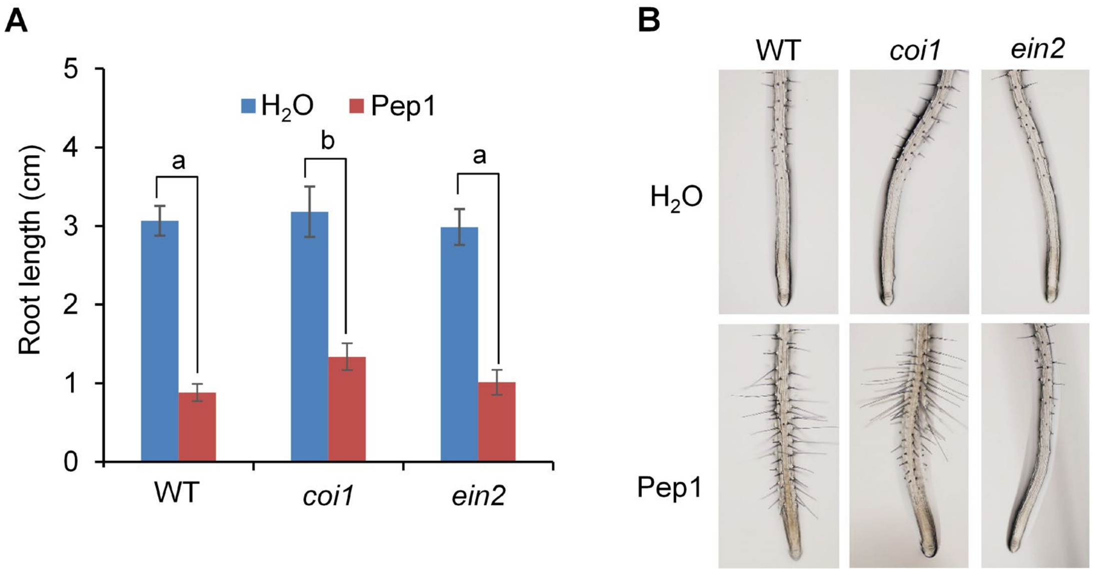

Supplementary figure 6. Pep1 suppresses root growth and induces root hair formation by acting on hormone signaling.

(A) JA but not ET signaling is required for Pep1 suppression on root growth. Three-day old plate-grown seedlings were transferred onto 1/2 MS plate containing 1 μM Pep1 for another five days before measurement of root length. Significant differences were shown by different letters (Student's *t*-test, $n \geq 8$).

(B) ET but not JA signaling is required for Pep1-induced root hair formation. Five-day old plate-grown seedlings were treated with 1 μM Pep1 on root tips, followed by imaging 24 hours later.

All experiments were repeated twice with similar results.

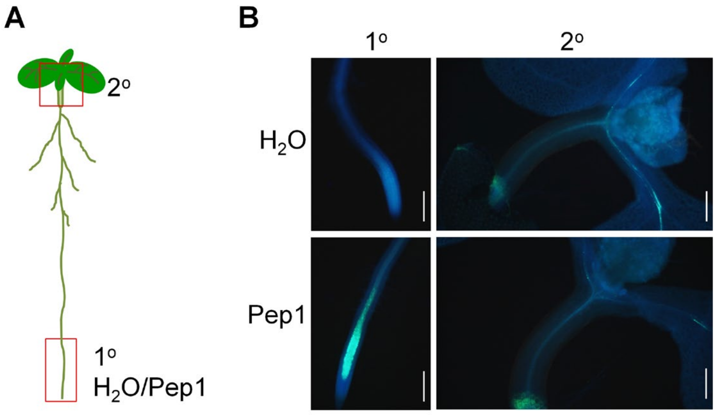

Supplementary figure 7. Pep1 application in root does not induce callose deposition in shoot. (A) A schematic diagram for the definition of local (1°) and systemic (2°) tissues (B) Pep1 induces local root callose deposition but not systemic shoot callose deposition. Root of one-week old plate-grown seedlings were treated with H_2O, or 1 μM Pep1 for 24 hours, followed by callose staining. The experiments were repeated three times with similar results. Bar = 200 μm.

Supplementary Table 1. Primers used in this study.

Gene name	Gene ID	Forward primer (5'→3')	Reverse primer (5'→3')
UBQ10	AT4G05320	AGATCCAGGACAAGGAAGGTATTC	CGCAGGACCAAGTGAAGAGTAG
PROPEP1	AT5G64900	GAGCTATCTTGAAATGCCTTGG	CTAACTTTCTCCTTCCCCCTTT
PROPEP2	AT5G64890	AAGACTATGGTGTTGCTCTGAA	ATCCCTTTTCTTTGATTTGGCC
PROPEP3	AT5G64905	ACGATTCCTCTCTTGAAGTGTT	GCTTAGTCTTGTTCTTCCCTCT
PEPR2	AT1G17750	CAATGAGTTTAGGCCTGAATCG	TATCTTCAAGTCTGACTCGCTC
VSP2	AT5G24770	TCAGTGACCGTTGGAAGTTGTG	GTTCGAACCATTAGGCTTCAATATG
PMR4	AT4G03550	CAAGCAATGAAGACTTCTACCG	ATTACTGAAGAAAGCAATCCGC
CYP81F2	AT5G57220	CTTCTTGCAGATTTGACGTTCA	TCCTTGTTATGAACCTGGTCTC
COMT1	AT5G54160	GGCTTTAAAATCCGCCTTAGAG	CAGGATTTTTGGTCGGAAGTTT
CCoAMT	AT1G67980	ATTGGTGAACGACAAATGTGAG	CCCCAACCTTCACCAATTTTAG
PR1	AT2G14610	CTAAGAGGCAACTGCAGACT	GTATGGCTTCTCGTTCACAT
PR5	AT1G75040	AGGATTTGAATTGACTCCAGGT	CCATCGCCTACTAGAGTGAATT
PDF1.2a	AT5G44420	CTTATCTTCGCTGCTCTTGTTC	TGGGAAGACATAGTTGCATGAT
PDF1.3	AT2G26020	CTCTTGTTCTCTTTGCAGCTTT	TGGGAAGACATAGTTGCAAGAT
ZAT12	AT5G59820	TGTCGTCTGGATTGATGAAGAA	GATTTCTTCAACGTAGTCACCG
